# Machine learning-based hyperspectral wavelength selection and classification of spider mite-infested cucumber leaves

**DOI:** 10.1007/s10493-024-00953-0

**Published:** 2024-08-23

**Authors:** Boris Mandrapa, Klaus Spohrer, Dominik Wuttke, Ute Ruttensperger, Christine Dieckhoff, Joachim Müller

**Affiliations:** 1 Institute of Agricultural Engineering, Tropics and Subtropics Group, University of Hohenheim, Stuttgart, Germany; 2 Wolution GmbH & Co. KG, Munich, Germany; 3 State Horticultural College and Research Institute, Heidelberg, Germany; 4 Center for Agricultural Technology Augustenberg, Karlsruhe, Germany

**Keywords:** Hyperspectral imaging, *T. urticae*, Effective wavelengths, Supervised machine learning, Feature selection

## Abstract

Two-spotted spider mite (*Tetranychus urticae*) is an important greenhouse pest. In cucumbers, heavy infestations lead to the complete loss of leaf assimilation surface, resulting in plant death. Symptoms caused by spider mite feeding alter the light reflection of leaves and could therefore be optically detected. Machine learning methods have already been employed to analyze spectral information in order to differentiate between healthy and spider mite-infested leaves of crops such as tomatoes or cotton. In this study, machine learning methods were applied to cucumbers. Hyperspectral data of leaves were recorded under controlled conditions. Effective wavelengths were identified using three feature selection methods. Subsequently, three supervised machine learning algorithms were used to classify healthy and spider mite-infested leaves. All combinations of feature selection and classification methods yielded accuracy of over 80%, even when using ten or five wavelengths. These results suggest that machine learning methods are a powerful tool for image-based detection of spider mites in cucumbers. In addition, due to the limited number of wavelengths, there is also substantial potential for practical application.

## Introduction

The two-spotted spider mite (*Tetranychus urticae* Koch) is a highly destructive pest that feeds on over 1150 plant species, including more than 150 crops (Migeon et al. 2010; Rioja et al. 2017; Van Leeuwen et al. 2010). It particularly affects cucumber plants in horticultural production, thriving in warm climatic conditions that favor its rapid development. Spider mites primarily feed on the abaxial side of the leaf surface by ingesting the contents of mesophyll cells (Park and Lee 2002). This leads to the loss of chlorophyll, the reduction of the photosynthetic rate, and the formation of chlorotic spots (Bensoussan et al. 2016), ultimately leading to leaf defoliation and crop losses (Santamaria et al. 2020).

Integrated pest management (IPM) is a strategy that aims to reduce the use of pesticides and thus minimize the disruption of agricultural ecosystems. A part of IPM is biological control, which includes the application of parasitoids, predators, pathogens, and biological pesticides (Debach 1964). Biological control is most effective when spider mite populations are low (Fraulo and Liburd 2007), making large-scale monitoring and early detection a prerequisite for successful IPM application (Lowe et al. 2017).

Biotic factors, such as pest infestations, can induce alterations in the physical and chemical characteristics of leaf tissue. These alterations can be detected through spectral methods. For example, spectral changes within the visible range are associated with the development of chlorotic spots caused by spider mite feeding, while changes in the range between 700 nm and 1300 nm are indicative of alterations in the mesophyll cell structure (Herrmann et al. 2017; Uygun et al. 2020). Additionally, Curran et al. (1990) suggested that the red edge spectral region (690–740 nm) could be responsive to changes in chlorophyll content.

Hyperspectral imaging (HSI) measures the spectral and spatial characteristics of an object by imaging it at numerous different wavelengths. HSI gives information across ranges of electromagnetic spectra, including the visible range (VIS, 400–700 nm) and the near-infrared range (NIR, 700–1000 nm), with the possibility of spectral steps below 1 nm (Mahlein et al. 2018). The visible range can be subdivided into three spectral regions: blue (400–500 nm), green (500–600 nm), and red (600–700 nm) (Liu and van Iersel 2021). HSI is able to capture the spectral alterations in plant tissue induced by pests, thereby facilitating timely and effective IPM interventions.

Numerous studies have investigated the spectral detection of spider mite infestation in crop production. Fraulo et al. (2009) utilized diffused reflectance spectroscopy in order to distinguish spectral areas affected by spider mite feeding on strawberry leaflets and estimate the quantity of spider mite density. Maximum spectral alterations were detected in the green (around 500 nm), red (around 700 nm), and NIR (800–1300 nm) spectral regions. Lan et al. (2013) examined the spectral response of spider mite-infested cotton plants and found that four wavelengths (550 nm, 560 nm, 680 nm, and 740 nm) could differentiate between spectral variances among the plants treated with different dosages of the acaricide. Martin and Latheef (2017) demonstrated that a multispectral optical sensor, which generated spectral data for the calculation of NDVI (Normalized vegetation difference index) index, was able to discern various spider mite infestation levels on greenhouse cotton. NDVI was calculated with red (660 nm) and NIR (770 nm) data. Spectral information on greenhouse-grown pepper and bean leaves affected by spider mite feeding was researched by Herrmann et al. (2017). It was concluded that damage detection was possible by partial least squares discriminant analysis (PLS-DA). The classification accuracy with hyperspectral data reached 83.5% and 94.2% for pepper and bean, respectively, whereas it was at 73.1% and 67.3% with multispectral data, respectively. For the multispectral data, five wavelengths were utilized: 490 nm, 560 nm, 660 nm, 715 nm, and 790 nm. Huang et al. (2018) combined a multispectral camera with the reflectance values of three wavelengths (550 nm, 650 nm, and 800 nm) and a neural network classification method to successfully discover spider mite damage in cotton, reaching an overall accuracy of 95%. Using an RGB camera, Uygun et al. (2020) created an image-processing method for spider mite damage detection on cucumber leaves and compared it with visual expert observations. In addition, Nieuwenhuizen et al. (2020) reported that a combination of a multispectral camera and a high-resolution RGB camera could reveal spectral differences between healthy and infested tomato leaves, obtaining an accuracy of up to 90%. In the multispectral system, three wavelengths were used: 700 nm, 716 nm, and 747 nm. Gonzalez-Gonzalez et al. (2021) studied the ability of hyperspectral imaging for recognition of spider mite damage on citrus leaves under laboratory conditions. It was concluded that the method could successfully differentiate between undamaged and damaged upper leaf sides, with a damage detection rate of 100% when three wavelengths (670 nm, 700 nm, and 740 nm) are used. Finally, Aeberli et al. (2022) used distinctive machine learning classification models and variable selection methods to discriminate banana plants infested with spider mites from healthy plants by choosing effective wavelengths. The authors reported a prediction accuracy of up to 86% based on twenty selected wavelengths from different spectral regions.

Different approaches have been employed to detect spider mite infestation in various crops, with studies identifying specific wavelengths or combinations of wavelengths for this purpose. While research has demonstrated the potential of machine learning methods for classifying symptoms on plants caused by biotic factors, as well as the effectiveness of hyperspectral data compared to multispectral data for improving classification accuracy, these techniques have not yet been applied to detect spider mite infestation in cucumbers.

The objective of this study was to combine machine learning with hyperspectral data from cucumber plants and evaluate its potential performance in classifying healthy and spider mite-infested cucumber leaves. The study aimed to achieve two main objectives: first, to identify effective wavelengths within the visible and near-infrared (NIR) spectral regions using various feature selection techniques, and second, to determine the number of wavelengths required to achieve acceptable classification accuracy using different machine learning algorithms. Finally, the classification performance of all and selected effective wavelengths was compared.

## Materials and methods

### Plant material and treatments

**Fig. 1:**
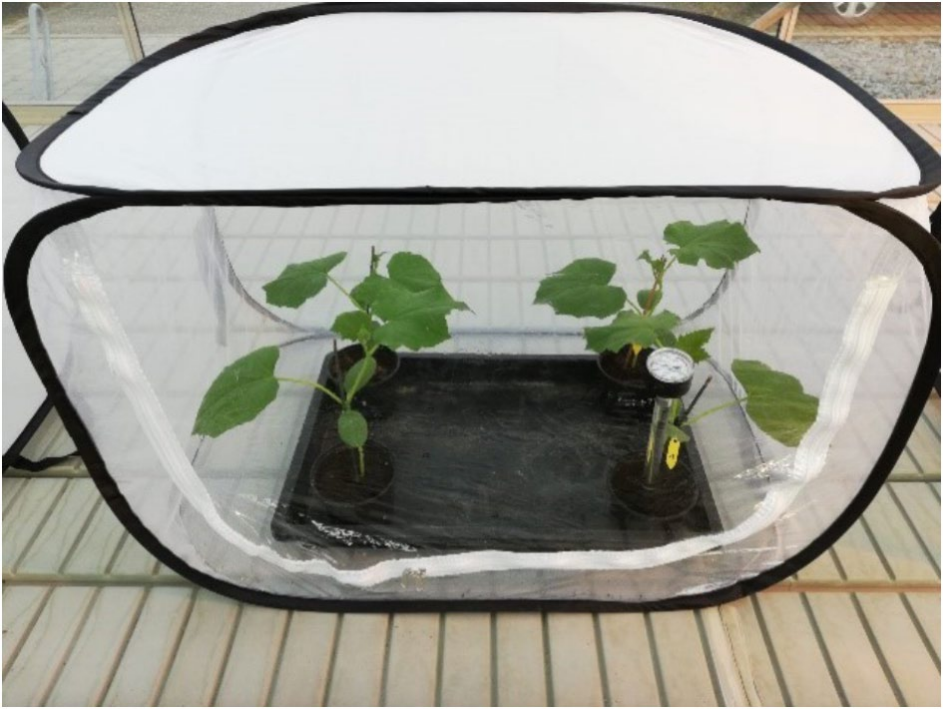
Transparent insect mesh cage with four cucumber plants and tensiometer

The study was conducted over 14 days, and twenty-eight cucumber plants (*Cucumis sativus* L.) were each sown in 12-cm-diameter pots with substrate (BP Substrat 2, Rez, 172, Klasmann-Deilmann, Geeste, Germany). All plants were grown under controlled conditions and were equally separated after three weeks into two treatments: healthy and infested. Fifty spider mites were placed on the first leaf of each cucumber plant in the infested group. The infestation was done under a stereomicroscope and with a fine brush. Afterwards, the pots were randomly placed inside transparent insect mesh cages (three or four per cage) with watering trays at the bottom and kept in a greenhouse (Fig. 1).

The plants were watered using tap water. For the purpose of monitoring soil water potential, tensiometers (Analog tensiometer, STEP Systems GmbH, Nuremberg, Germany) were installed in the substrate. Soil water potential was kept above − 150 hPa to avoid drought stress. One tensiometer was installed in every cage. Using a data logger (UHADO-16, LogTag, Auckland, New Zealand), temperature and humidity data were simultaneously recorded on an hourly basis. The temperature ranged from 20.4 °C to 34.4 °C during the experimental period, while the corresponding relative humidity varied from 27.6 to 82.3%. Natural sunlight was supplemented by high-pressure sodium lamps (MASTER SON-T PIA Agro 400 W, DHLicht GmbH, Wülfrath, Germany) with a switching point of 20 klx and an exposure duration of 7 a.m. to 7 p.m.

### Imaging sensor and image acquisition

**Fig. 2:**
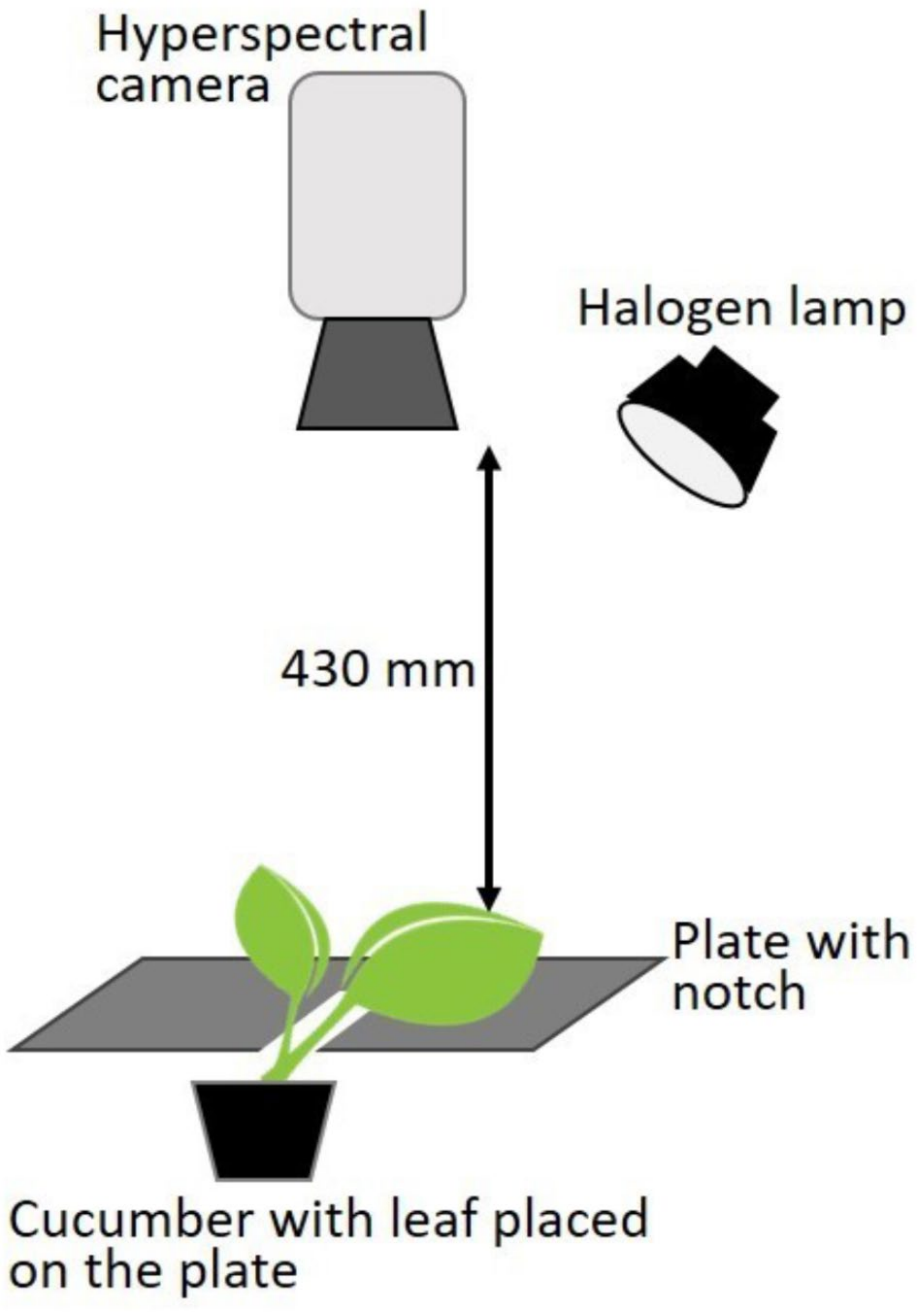
Sketch of imaging setup with hyperspectral camera

Reflectance data were collected indoors with a snapshot hyperspectral camera (UHD 185 Firefly, Cubert GmbH, Ulm, Germany). In this study, the range between 502 and 950 nm was used with a spectral step of 4 nm. The camera simultaneously captures panchromatic (grey) images (1000 × 1000) and spectral cubes (50 × 50 × 113), after which its software applies a pan-sharpening method to create final 3D cubes. These cubes represent three-dimensional matrices, where the first two dimensions (x, y) are spatial coordinates and the third dimension (z) represents the respective wavelengths. Prior to each data collection, an exposure time had to be set and a radiometric calibration executed. This was done after switching on a lamp (HL 120SA, Smartwares, BH Tilburg, The Netherlands) with a 120 W halogen bulb (Osram GmbH, Munich, Germany) installed next to the camera. The exposure time was set to automatic mode and varied between 6 and 7 ms throughout the study. Furthermore, the radiometric calibration included white and dark reference corrections available in the camera’s software. The white correction was acquired with a white reference panel provided by the manufacturer. The dark correction was realized by covering the lens with its cap. The distance between the lens and the white reference panel, or cucumber leaves, was 430 mm. After the radiometric calibration, cucumber leaves remaining on the plant were placed on a fixed black plate under the camera, and snapshots of the upper side of the leaves were captured. During the experiment, healthy leaves were captured first, followed by infested leaves. A notch was created so that the leaves could be positioned in the middle of the plate, creating a 90° angle between the camera lens and the observed leaf tissue (Fig. 2). Having finished data compilation, the plants were returned to the cages in the greenhouse. Hyperspectral snapshots were taken daily, converted to ENVI format, and exported to an external drive for further processing. In total, 235 snapshots of healthy leaves and 279 pictures of infested leaves were captured. To avoid bias towards the majority class, data sets of the same size with images of healthy and visibly infested leaves were used for further work. Hence, 440 snapshots were randomly chosen, 220 from each group. Additionally, RGB images of the cucumber leaves were captured to document visible symptom development during the experiment.

### Hyperspectral image pretreatment

Data pretreatment was completed in Python using the cloud-based Google Colaboratory platform (Colab) and Unscrambler 11 (Camo Analytics AS, Oslo, Norway). Python libraries such as spectral, drive, numpy, pandas, os, cv2, and sklearn were used (*Google Colaboratory*, n.d.). The initial step for the Python procedure was to execute segmentation between leaves and background. The segmentation was achieved by forming a binary mask and subsequently thresholding the images with the normalized vegetation difference index (NDVI) (Rouse et al. 1974). To eliminate pixels with mixed spectral information on the leaf edges, a morphological operation (erosion with a 3 × 3 matrix) was applied and the mixed pixels were excluded from further analysis. The NDVI thresholding and the erosion mask operations formed regions of interest (ROI). The segmentation line represents the borders of ROI. Once an image was segmented, the reflectance values of all pixels in each ROI were smoothed, averaged, and a mean value was created for each wavelength. This resulted in the mean raw spectral signature (MRSS) for 113 wavelengths per leaf.

The final step of data preprocessing included the extraction and transfer of all spectral signatures to Unscrambler 11. Unscrambler 11 is a software used for multivariate spectroscopic data analysis. A chemometric spectral technique called standard normal variate (SNV) was chosen to remove scattering effects from the averaged spectra. SNV transforms the spectra by subtracting their means and dividing them by their standard deviations (Mishra et al. 2020), resulting in mean normalized spectral signatures (MNSS).

### Effective wavelength selection and machine learning algorithms

Hyperspectral information is represented by various variables (wavelengths), but multicollinearity and redundancy can pose challenges in subsequent data analysis. To solve these issues and reduce the number of variables necessary for the successful classification of distinctive treatments, the selection of effective wavelengths is essential. This was completed using three feature selection methods available in the scikit-learn library: feature importance (FI), select k-best (SKB), and select percentile (SP) (Garreta and Moncecchi 2013). FI is a technique that determines the importance of each feature by assigning a score based on its contribution to the predictive power of the model. On the other hand, SKB and SP methods identify the top-performing features based on their statistical relationship with the target variable. Using FI, SKB, and SP, both the ten best and five best wavelengths were selected. Thereafter, the suitability of all wavelengths, in addition to the selected wavelengths, to accurately classify healthy and infested cucumber leaves was evaluated. This was conducted with three machine learning algorithms, namely random forest (RF), support vector machine (SVM), and k-nearest neighbor (KNN) (Garreta and Moncecchi 2013). Hyperparameter tuning and five-cross validation were performed to determine the best possible parameters for machine learning algorithms. Table 1 gives an overview of parameters selected for each machine learning algorithm. After that, combinations of feature selection methods and machine learning models were chosen. This was done based on the pretesting conducted to achieve the best possible classification accuracy. In the end, FI-selected wavelengths were used with RF, SKB-selected wavelengths were used with SVM, and SP-selected wavelengths were used with KNN.

**Table 1:** Machine learning algorithms and respective parameters utilized in this study

Algorithm	Description	Parameters
RF	Ensemble learning method that links multiple decision trees to make accurate predictions by employing feature importance	n_estimators = 100 max_depth=; min_samples_split = 5 min_samples_leaf = 1 class_weight = balanced
SVM	Algorithm that opts for an optimal hyperplane to distinguish and categorize data points based on their proximity to different classes	C = 10 gamma = 1 kernel = rbf
KNN	Non-parametric algorithm that allocates labels to new data points based on the class of their nearest neighbors in the training dataset	n_neighbors = 5 weights = uniform

Samples were randomly split, and the ratio of training to testing data was 70:30. The metrics used to assess RF, SVM, and KNN algorithms were accuracy, precision, recall, and F1 score (Table 2). Accuracy provides an overall measure of correctness, precision focuses on the accuracy of positive predictions, recall focuses on the model’s ability to capture all positive instances, and the F1 score combines precision and recall to provide a balanced evaluation of a model’s performance.

**Table 2:** Machine learning metrics

Name	Formula	Description
Accuracy	(TP + TN) / (TP + TN + FP + FN) * 100	Percentage of correctly classified samples made by the model
Precision	TP / (TP + FP) * 100	Percentage of true positive predictions made by the model
Recall	TP / (TP + FN) * 100	Percentage of true positive samples that were correctly predicted by the model
F1 score	2 * Recall * Precision / (Recall + Precision)	Harmonic mean of recall and precision

## Results

### Temporal development of infestation symptoms and data preprocessing

**Fig. 3:**
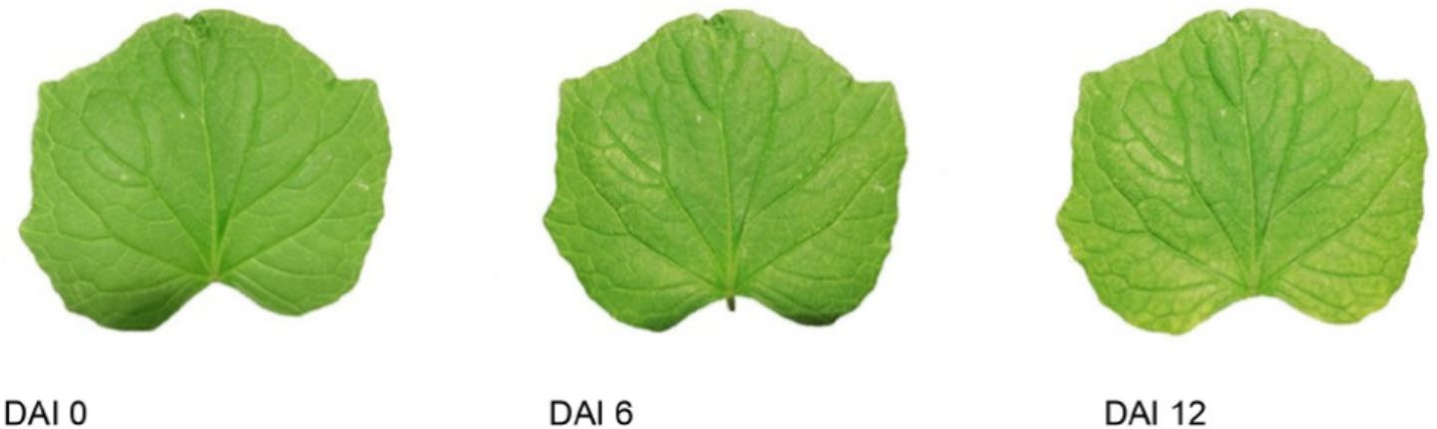
Development of spider mite infestation symptoms on a cucumber leaf at different days after infestation (DAI)

Spider mite infestation symptoms increased steadily during the experiment. Figure 3 exemplifies RGB images of the same cucumber leaf and illustrates how the green color progressively decreased over time. In some cases, the first visible symptoms of infestation appeared as early as one day after spider mites had been placed on experimental plants. It was also observed that the visible infestation symptoms were not evenly distributed across the leaf but occurred in local hotspots that increased throughout the experiment.

**Fig. 4:**
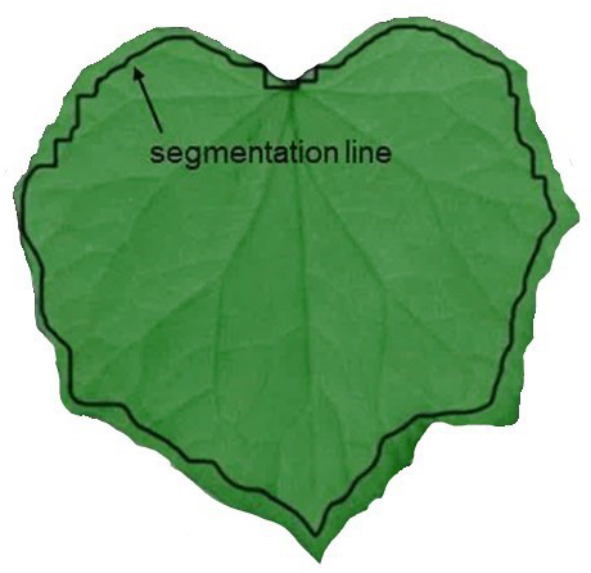
Identification of leaf pixels in ROI based on dual mask approach (NDVI mask and erosion mask)

ROI was created for each leaf in order to process spectral leaf information. Figure 4 illustrates one leaf and the segmentation line used for this purpose, which separates the ROI pixels from the unwanted pixels in the surrounding area. It is also evident that the ROI is smaller than the real leaf area. This reflects the impact of the applied segmentation process, which in the first step identifies all leaf pixels by means of the NDVI mask and in the second step excludes the leaf edge (mixed) pixels with the erosion mask.

**Fig. 5:**
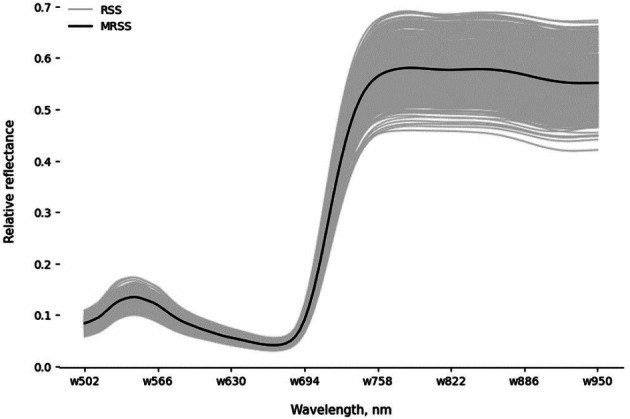
Raw spectral signatures (RSS) of single pixels in ROI of an exemplary leaf (grey lines) and mean raw spectral signature (MRSS) (black line)

Depending on the size of the leaf, the number of pixels within a single ROI varied between approximately 150,000 and 300,000. For each single pixel, a raw spectral signature (RSS), namely the light reflection line along 113 measured wavelengths between 502 and 950 nm, could be obtained. Figure 5 demonstrates the variation of all raw spectral signatures (RSS, gray lines) in the ROI of one cucumber leaf, as well as their mean raw spectral signature (MRSS, black line). All RSS and MRSS showed a local maximum in the green spectral region, while reflection in the red spectral region dropped. In the non-visible spectral region, a strong increase in the reflectance data was observed between 690 and 740 nm. This region (red edge) is at the border of the near-infrared, where the visible spectral region ends and the NIR starts. The figure also illustrates that light in the NIR above 740 nm was strongly reflected from plant tissue. It is also evident that the measured differences in light reflection between single pixels were greatest in this spectral region.

**Fig. 6:**
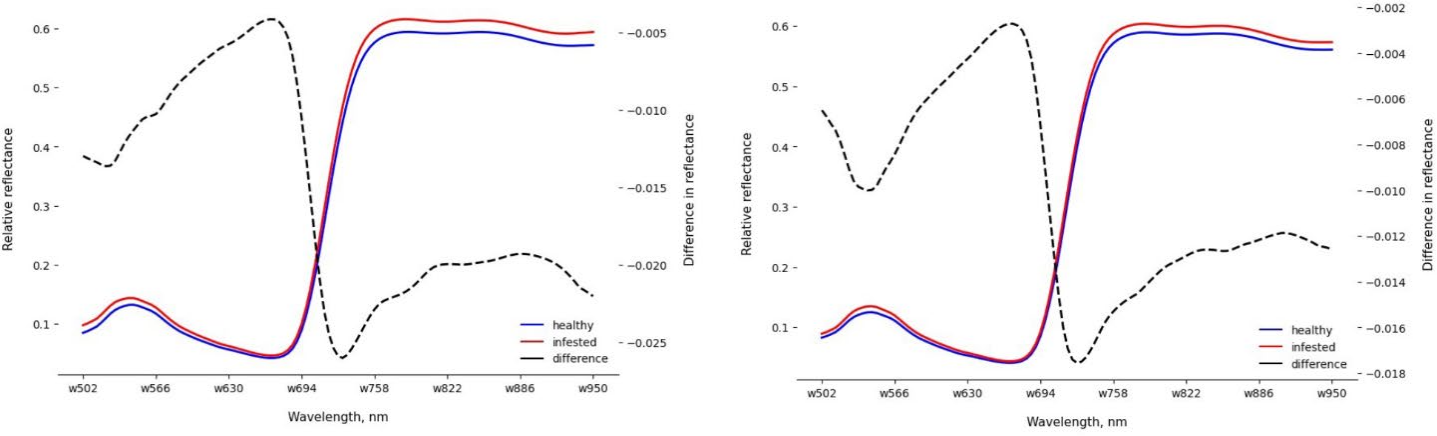
Averaged MRSS of healthy and infested cucumber leaves at 6 DAI (up) and 12 DAI (down)

Figure 6 depicts the averaged MRSS of healthy and infested cucumber leaves at 6 DAI and 12 DAI. The spectral signatures of infested leaves show higher reflectance values compared to healthy leaves, particularly evident in the green and NIR spectral regions. At 6 DAI, the reflectance difference between healthy and infested leaves ranges from 0.002 to 0.018 on the right y-axis, indicating spectral changes due to infestation. Over time, these differences became more pronounced, ranging from 0.005 to 0.025 at 12 DAI. This increase suggests a progressive alteration in leaf reflectance as infestation persisted.

**Fig. 7:**
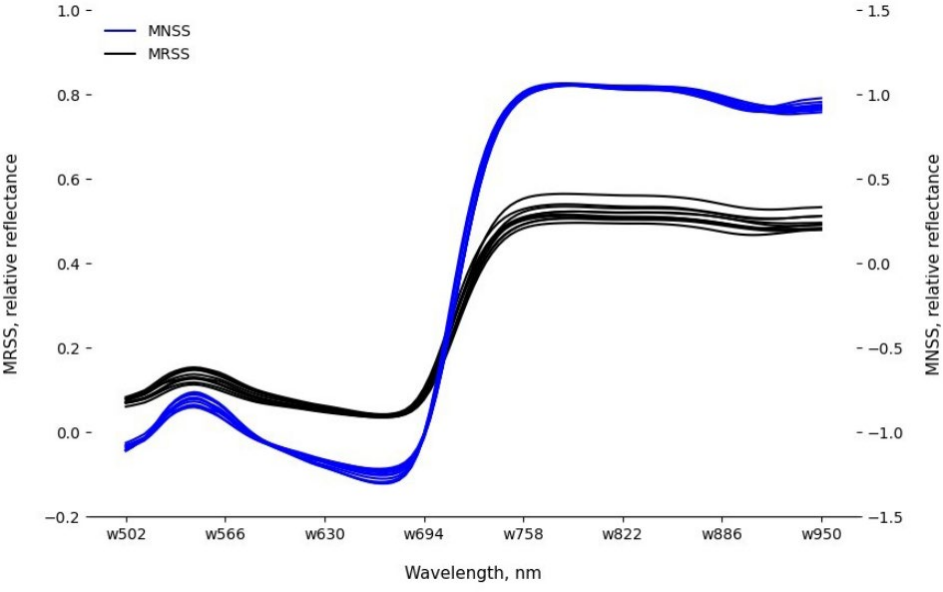
Twenty randomly selected mean raw spectral signatures (MRSS, left axis) and mean normalized spectral signatures (MNSS, right axis)

Thereafter, MRSS were used to classify two treatments based on spectral differences caused by spider mite infestation. To be able to compare all images of healthy and infested leaves over time, MRSS were normalized in the final preprocessing step, and mean normalized spectral signatures (MNSS) were created (Fig. 7). It is apparent that the normalization reduced differences between spectral signatures in the NIR, while those in the green spectral region were maintained. Furthermore, the normalized spectral signatures showed more heterogeneity in the red spectral region when compared to the raw spectral signatures.

### Effective wavelengths selection

**Fig. 8:**
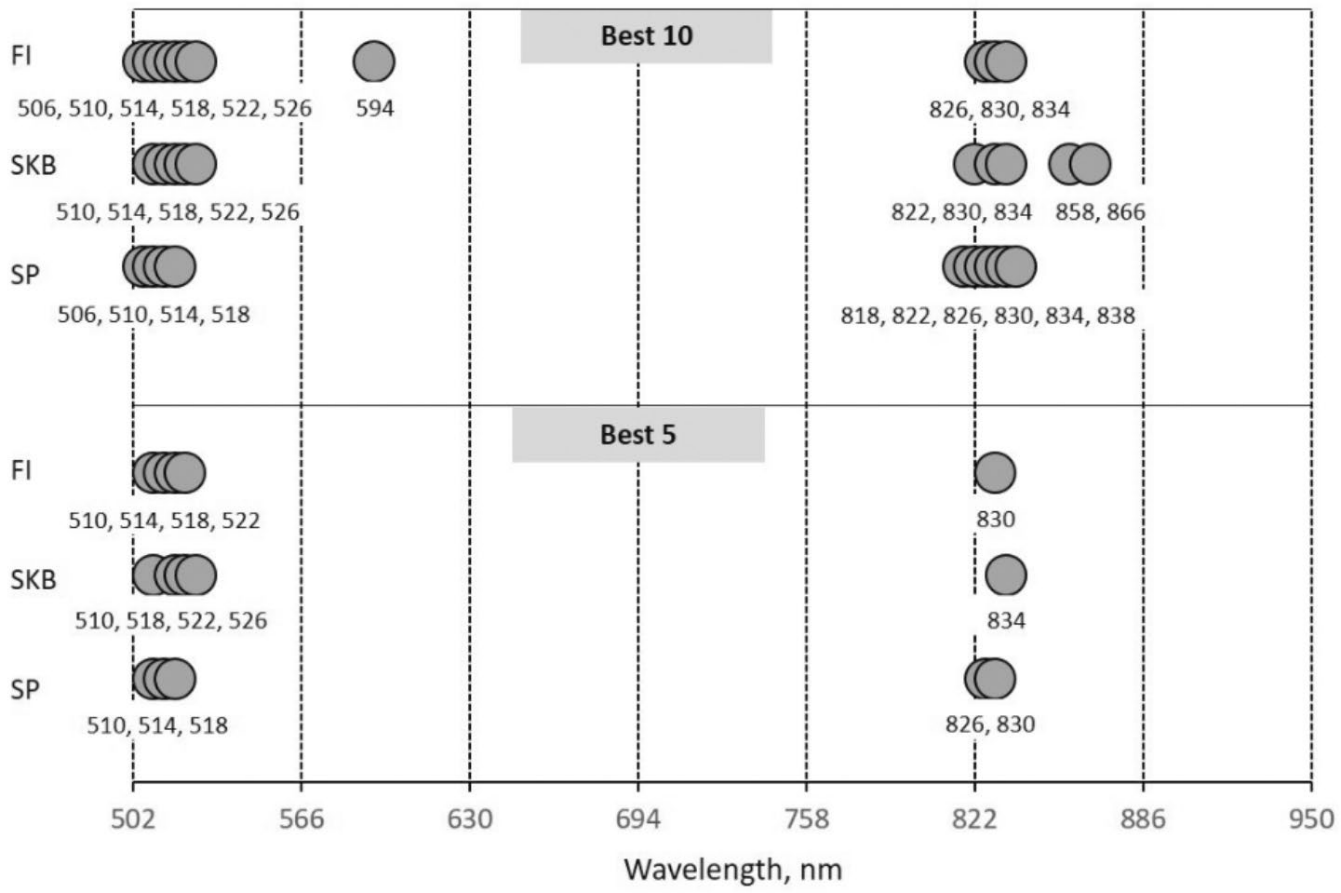
Effective wavelengths selected by feature selection methods

Figure 8 depicts the effective wavelengths obtained through three different feature selection methods: FI, SKB, and SP. It is noticeable that all wavelengths come from two spectral regions: green and NIR. Among the ten most effective wavelengths, the FI method selected seven from the green spectral region and three from the NIR. The SKB method chose an equal number of wavelengths from both spectral regions, with five wavelengths each from the green and NIR spectral regions. Alternatively, the SP method preferred four wavelengths from the green spectral region and six wavelengths from the NIR. Upon closer examination of the five most effective wavelengths, both the FI and SKB methods opted for four wavelengths from the green spectral region and one from the NIR. However, the SP method chose three wavelengths from the green spectral region and two from the NIR. Interestingly, regardless of the number of wavelengths considered, two common wavelengths emerged across all feature selection methods: 510 nm and 518 nm.

### Classification analysis results

**Fig. 9:**
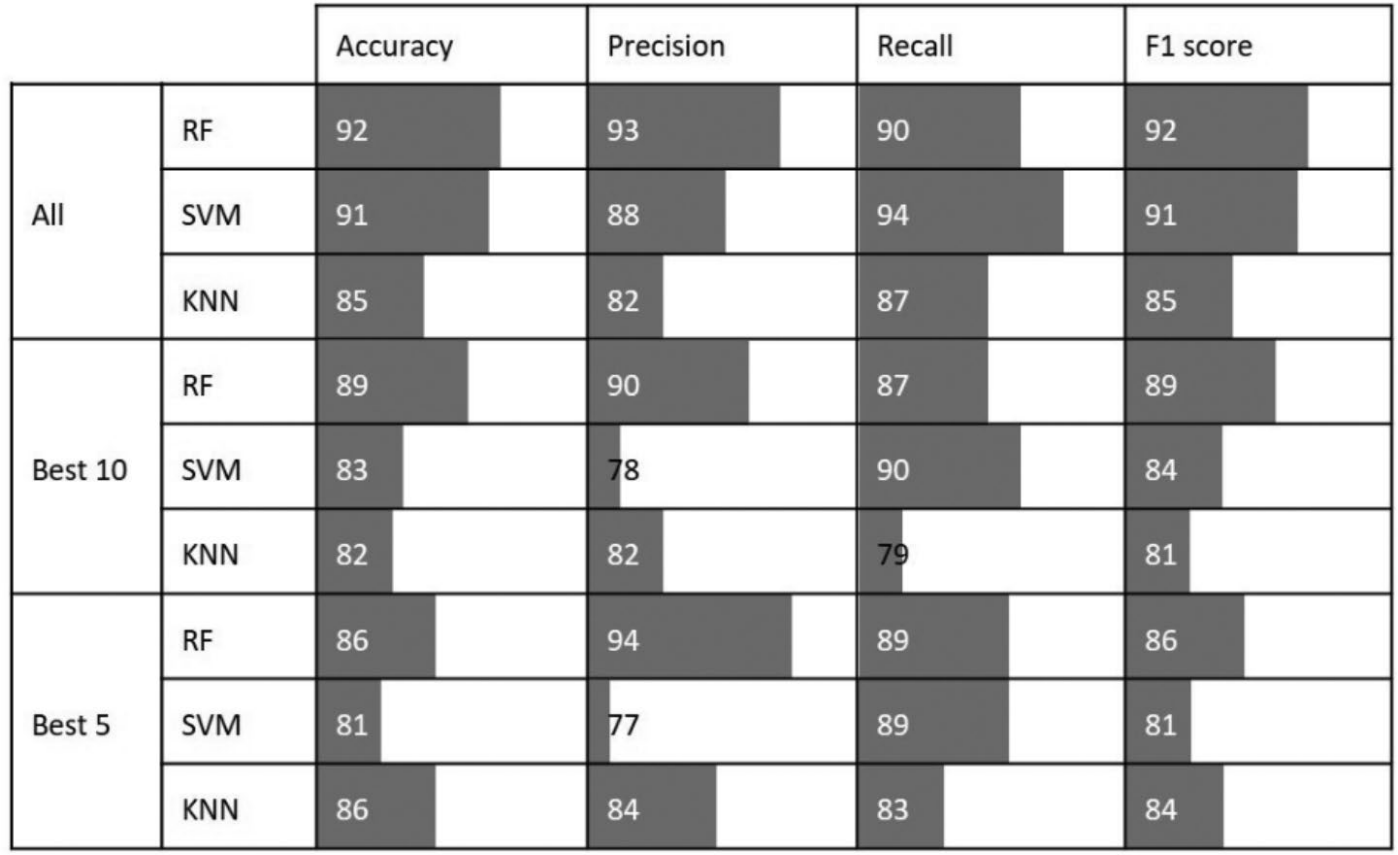
Model comparison with all, ten best and five best wavelengths

Figure 9 shows the classification performance of healthy and spider mite-infested cucumber leaves by using RF, SVM, and KNN models. This was evaluated with accuracy, precision, recall, and the F1 score. Notably, the RF model demonstrated superior performance, surpassing the other models with metrics consistently above 90% with the full spectrum. This model achieved the highest accuracy, precision, and F1 score values, those being 92%, 93%, and 92%, respectively. The SVM model also exhibited excellent performance, having the highest recall value with all wavelengths, at 94%, but it obtained a slightly lower precision value, which was at 88%. Finally, the metrics for the KNN model ranged between 82% (precision) and 87% (recall) 0.

In the case of ten best wavelengths, the RF model excelled over the other models, as its accuracy (89%), precision (90%), and F1 score (89%) values were the highest. The model built with SVM reached the highest recall value at 90%, but its precision value was at 78%. Conversely, the precision value of the KNN-built model was higher than that of the SVM model, being at 82%, whereas accuracy, recall, and F1 score values were at 82%, 79%, and 81%, respectively.

With five best wavelengths, the RF model achieved the highest precision and F1 score metrics, having the values of 94% and 86%, respectively. Both the RF and SVM models achieved a recall value of 89%, while the RF and KNN models’ accuracy value was at 86%. Interestingly, the KNN model demonstrated the highest accuracy and precision among its three models.

The RF model consistently demonstrated superior performance across three figures, highlighting its effectiveness in classifying healthy and spider mite-infested cucumber leaves. Nonetheless, the SVM and KNN models also exhibited strengths in specific metrics, such as recall and accuracy, respectively.

## Discussion

### Development of infestation symptoms and spectral signatures

Symptoms caused by the feeding of spider mites on the underside of cucumber leaves, such as chlorotic spots recognizable by a yellow discoloration, were observed on most of the leaves already one day after infestation. The reason for the color change was the loss of pigment. With intensified feeding, the yellow discoloration became more obvious, resulting in the reduction of green leaf tissue. This yellow discoloration caused increased reflectance values of infested cucumber leaves, which is illustrated in Fig. 6. The spectral signatures derived from the hyperspectral imaging of cucumber leaves showed typical leaf reflectance data. In the visible spectral region (502–700 nm), leaf reflectance was low, whereas it was high in the NIR spectral region. Decreased leaf reflectance in the visible spectral region is linked to photosynthetic pigments, mainly chlorophylls and carotenoids (Peñuelas and Filella 1998). A local maximum was observed in the green spectral region, indicating the green portion of visible light that was not absorbed (Rock et al. 1986). In contrast, the absorption around 680 nm is related to the red chlorophyll absorption band (Bauriegel et al. 2011). An increased slope in the spectral signatures beginning between 690 nm and 740 nm indicates the transition between strong absorbance and strong reflectance, known as the red edge region, which is relevant to the chlorophyll content (Pen Uelas et al. 1995; Roy 1989).

The differences in intensities of single pixels of a leaf are presented in Fig. 5. This can be attributed to the illumination variation caused by the lighting system used in the experiment (Mishra 2020). After the preprocessing steps, these differences were less noticeable in the NIR spectral region but remained in the visible spectral region. A possible clarification might be that, despite the fact that all spectral signatures in the figure were established from healthy cucumber leaves, inequalities in the quantity of pigments present in the green and red spectra are still possible since it is not to be expected that all leaves have identical spectral signatures.

Hyperspectral data from the same set of cucumber leaves over 14 days were collected. This repeated imaging method could introduce pseudo-replication, where non-independent measurements are treated as independent, potentially biasing the results. However, since plant leaves grew and changed over time, their spectral profiles also evolved. Therefore, each spectral signature was independently considered, given the changes in the plant leaves over the study period. Although data from the same plants were randomly split into both training and testing sets, this approach may have introduced some degree of correlation between training and testing data. To mitigate this risk and ensure the robustness of our machine learning analysis, snapshots for our study were randomly selected, maintaining an equal representation of healthy and infested leaves. While this method did not completely eliminate the risk of pseudo-replication, it allowed us to leverage the full temporal variability in the spectral profiles and identify effective spectral wavelengths for distinguishing between healthy and infested leaves.

### Feature selection and machine learning models

Figure 7 shows variations in the red spectral region after applying SNV to the raw spectral data. Despite this, our feature selection methods (FI, SKB, SP) did not identify red-region wavelengths as relevant for the classification of healthy and spider mite-infested cucumber leaves. The ten best and five best selected wavelengths in this study are exclusively from the green and NIR spectral regions. Several reasons may have led to this outcome. In a study by Fraulo et al. (2009), the importance of the green spectral range was emphasized, among others, as spider mites feed on cells containing chloroplasts. Concerning the NIR, several studies confirmed the responsiveness of wavelengths from this spectral region towards damage caused by biotic factors. For instance, Reisig and Godfrey (2007) identified the 850 nm wavelength as significant for discriminating between insect-infested and healthy cotton leaves. In a publication by Prabhakar et al. (2012), it was concluded that leaf cell structure collapses under biotic stress, and it was captured by the wavelengths from the NIR spectral region. No wavelengths from the red spectral region were selected by the feature selection methods. This lower importance of the red wavelengths was confirmed by additional testing. Modeling results with all 22 available wavelengths from the red spectral region yielded accuracies between 60% and 70%, while “only” five wavelengths outside this region achieved accuracies of up to 86% (Fig. 9).

**Fig. 10:**
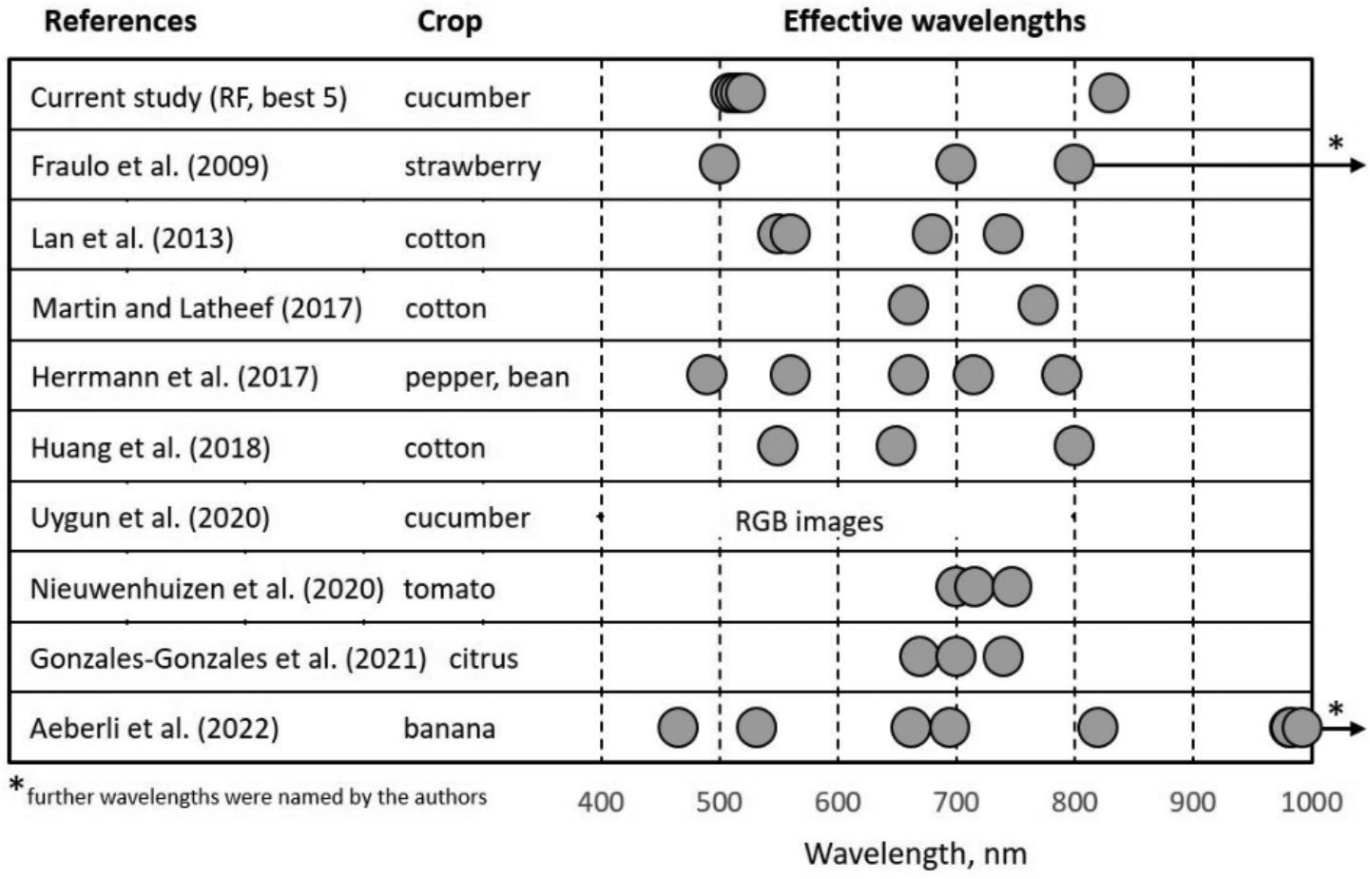
Overview of studies investigating spectral imaging for detection of spider mite damage

Figure 10 compares the results of this study with publications mentioned in the introduction, in which the symptoms of leaf damage caused by spider mites were investigated using spectral analysis of plant tissue. It is interesting that wavelengths from the red spectral region were selected as relevant in the majority of these studies. In general, the range of possible effective wavelengths is very large and includes the green, red, red edge, and NIR spectral regions. It can also be seen that different wavelengths are important for different crops. However, since different wavelengths were also selected for the same crop, the simple assumption of crop-dependent wavelengths cannot be supported. It is more likely that the effective wavelengths chosen in each specific study were dependent on the study objective (e.g., number of wavelengths), the experimental setup, the imaging sensors (RGB, multispectral, hyperspectral), and the data processing. In addition, other parameters, such as the prevailing light conditions (laboratory, greenhouse, outdoor), can influence the selection of wavelengths. Finally, the fact that no wavelengths from the red and red edge spectral regions were chosen in this study may also be due to the fact that feature selection methods emphasize predictive power in the selection of the most informative variables (Guyon 2003).

Using three distinctive supervised machine learning algorithms, the efficacy of selected wavelengths for accurate classification of healthy cucumber leaves and leaves with spider mite symptoms was evaluated. In three scenarios involving 113, 10, and 5 wavelengths, respectively, the RF model achieved better metrics than the SVM and KNN models. This might be due to the fact that RF is an ensemble method that is robust to noisy data and capable of capturing nonlinear relationships between spectral variables (Menze et al. 2009). A further advantage of RF over SVM and KNN is its ability to inherently identify important features, thereby enhancing predictive ability and model precision by concentrating on relevant wavelengths (Ali et al. 2012). In contrast, SVM and KNN models required auxiliary feature selection methods, SKB and SP, in order to test their classification ability with the reduced number of wavelengths. It should be noted that the KNN model had its leading performance with the five best wavelengths. One reason for this might be the curse of dimensionality, as the distance calculations between data points become more accurate with fewer variables, leading the KNN model to more precise measurements of the proximity of observations (Cunningham and Delany 2022; López-Serrano et al. 2016). Nevertheless, even with the reduced number of wavelengths, all models reached accuracies above 80%, which is consistent with other studies about spectral spider mite detection. With the use of multispectral data, Herrmann et al. (2017) reported 73.1% and 67.3% accuracy for healthy and spider mite-infested pepper and bean leaves, respectively. According to the same study, when hyperspectral data was used, accuracy was over 83.5% and 94.2% for pepper and bean leaves, respectively. In a study conducted by Aeberli et al. (2022), it was concluded that the accuracy of a KNN model for the classification of healthy and mite-infested banana leaves was up to 86% when carried out with the full spectral range (350–2500 nm). Having used 20 key wavelengths, the accuracy varied between 81% and 86%, depending on the wavelength’s selection method used. These results are similar to those in our research; however, it must be mentioned that the number of key wavelengths used in the abovementioned study was 20 and that the measurements were done at four different locations on the chosen leaves.

**Fig. 11:**
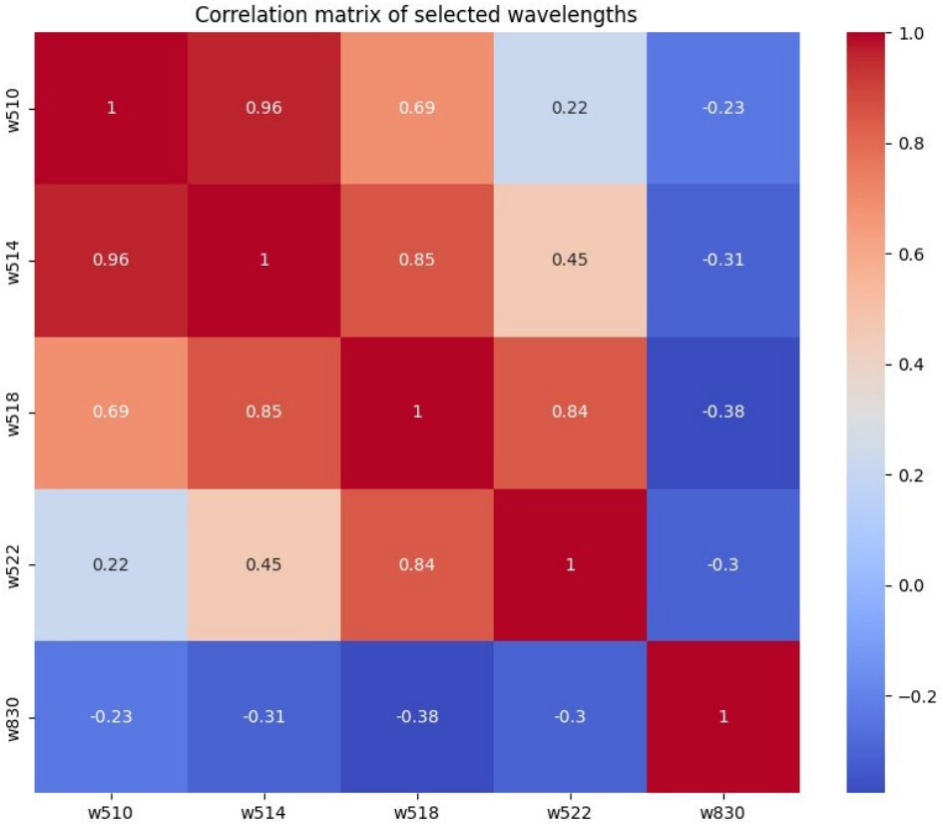
Correlation matrix of the selected wavelengths for RF (best 5)

In contrast to the reference studies, the effective wavelengths selected here are next to each other, which raises the question of redundant information. Figure 11 shows an example of the correlation matrix of the selected wavelengths for RF (best 5). Correlations of up to 0.96 suggest a redundancy among some wavelengths. Consequently, control tests were performed to address this potential redundancy, and likely redundant wavelengths were removed. For instance, wavelength 514 nm (w514) was explicitly deleted. However, this deletion resulted in lower accuracy (dropping from 86 to 82%), even when the neighboring wavelength was given greater weighting. Based on these results, no definitive conclusions should be drawn regarding the importance or redundancy of individual wavelengths. Instead, the close spacing of wavelengths should be interpreted as an indication that the corresponding spectral range is crucial for the classification. The high correlation suggests that these wavelengths collectively capture important features necessary for accurate classification.

Our study marks the initial exploration into the selection of effective wavelengths and their application for classifying healthy and infested cucumber leaves using machine learning techniques. However, these investigations were conducted under controlled conditions. It is necessary to extend this research to practical greenhouse environments, where several disturbing factors are present. Transitioning to the greenhouse setting introduces additional noise and variability. This transition can be supplemented and supported by the structured introduction of noise. According to Nansen et al. (2022), conducting sensitivity analyses by introducing noise into datasets can reveal its impact on classification performance. Thus, the sensitivity of models to noise and their robustness at different noise levels could be evaluated, especially since our models, based on 440 spectral signatures, may currently lack the necessary robustness to effectively mitigate the effects of noise. It is believed that larger data sets could improve the resilience of the model by capturing a wider range of disturbing influences. In addition, exploring noise filtering techniques could be a possible strategy to improve model reliability.

## Conclusion

Hyperspectral imaging was carried out under controlled conditions in the laboratory, and almost all of the examined leaves exhibited visible spider mite symptoms shortly after the start of infestation. Under these conditions, the investigated combined approaches of feature selection and supervised machine learning algorithms proved to be powerful methods for image-based spider mite detection.

Future research should aim to deepen and expand the investigations. Experimental limitations (e.g., due to pseudo-replication) need to be further investigated with different plants for training and testing, ensuring complete independence between these datasets.

It was striking that the effective wavelengths determined with the three different feature selection approaches were derived from the identical spectral regions (green, NIR). It was also striking that the investigated machine learning algorithms for the classification of spider mite infestation all showed satisfactory results with both ten and five selected wavelengths.

Conclusively, not all wavelengths in the investigated range between 502 nm and 950 nm were decisive for good classification accuracy. In addition, the number of wavelengths required could be significantly limited to less than ten.

There is a likelihood that similarly good or even better results could be achieved with alternative feature selection and classification algorithm combinations. Subsequent investigations will be aimed at confirming this. Future work will also focus on the verification of the identified wavelengths, on the further reduction of the required number of wavelengths, and on the early detection of spider mite infestation when no visible symptoms are present. Finally, these methods must be validated and optimized under practical greenhouse conditions.

## Data Availability

We declare all data is being provided within this manuscript.
